# Predictors of hospital stay in normotensive acute pulmonary embolism: a retrospective pilot study

**DOI:** 10.1080/20009666.2018.1466602

**Published:** 2018-06-12

**Authors:** Osama Mukhtar, Oday Alhafidh, Mazin Khalid, Jaspreet Kaler, Ebad Rahman, Binav Shrestha, Manal Bakhiet, Sumit Dahal, Bikash Bhattarai, Praveen Datar, Omar Abdulfattah, Vijay Gayam, Joseph Quist, Danilo Enriquez, Frances Schmidt

**Affiliations:** a Department of Medicine, Interfaith Medical Center, Brooklyn, USA; b Department of Pulmonology, Interfaith Medical Center, Brooklyn, USA

**Keywords:** Pulmonary embolism, severity index, prognosis, risk stratification, hospital stay

## Abstract

**Introduction**: The aim of our study is to determine the clinical, biochemical, and imaging factors that affect the duration of hospital stay in patients admitted with normotensive acute pulmonary embolism.

**Methods**: This was a single-center retrospective study conducted in a community hospital in New York metropolitan area for patients admitted from October 2015 to October 2017.

**Results**: A total of 79 patients were included, the mean age was 55.76 (*SD* = 17.33), 29 cases were males (37%) and 50 cases were females (63%). Among all patients, 17 cases had short length of stay (LOS) (≤2 days) and 62 cases had long LOS (>2 days). There were statistically significant differences in age (*p* = .041), presence of lung disease (*p* = .036), number of comorbidities (*p* = .043), and pulmonary embolism severity index (PESI) scores (original and simplified; *p* = .002 and .001, respectively). Logistic regression analysis showed that PESI score significantly predicted long LOS (*OR* 1.067, 95% *CI* [1.001, 1.137], *p* = .048). Similarly, sPESI significantly predicted long LOS (*OR* 0.223, 95% *CI* [0.050, 0.999], *p* = .050). Both regression models were adjusted for age, lung disease, and number of comorbidities.

**Conclusion**: Both original and simplified PESI scores were statistically significant predictors of duration of hospital stay. Patients with multiple comorbidities or with chronic lung disease were also likely to have prolonged hospital stay. None of the cardiac biomarkers affected the duration of hospital stay, neither did the presence of right ventricular dysfunction nor treatment modality.

## 1. Introduction

Acute pulmonary embolism (PE) is a leading cause of cardiovascular mortality worldwide; it is the third most frequent acute cardiovascular disease after acute coronary syndrome and stroke [1], with an incidence of 112 cases per 100,000 [2]. The aggregate cost estimation for newly diagnosed venous thromboembolism (VTE) in the USA is 7–10 billion dollars annually [3]. The mortality rate for untreated acute PE is as high as 30%, which drops to 8% if timely diagnosed and appropriately managed [4].

Patients with acute PE are commonly admitted to US hospitals for risk stratification and initiation of anticoagulation therapy [5]. Several risk stratification tools have been developed and implemented to help predict mortality in acute PE. Among the common prognostic scores are pulmonary embolism severity index (PESI) (original and simplified), Bova score, and Geneva prognostic score (GPS) [6–9]. Current recommendations suggest that low-risk PE (LRPE) patients are suitable for treatment out of the hospital or they can be treated as in-patients but to be discharged early [10]. The aim of this study is to evaluate these ‘mortality scores’ as predictors of early hospital discharge for patients who are being admitted for acute PE.

## 2. Patients and methods

### 2.1. Study design

We retrospectively reviewed electronic medical records of patients who presented to the emergency department of a single community hospital in Metropolitan Area in New York state and admitted with a diagnosis of acute PE (International Classification of Diseases, 10th Revision, ICD-10 code: I26) in the period from October 2015 to October 2017. Only patients who were normotensive on admission were included in this study. Patients who developed PE during hospitalization were excluded. The study protocol was approved by Institutional Review Board (IRB) prior to data collection. Informed consent was waived due to the retrospective nature of the study.

### 2.2. Patient selection

Patients were included in the study if they (1) were ≥18 years of age, (2) had at least one admission with acute PE confirmed by pulmonary CT angiography (CTA) or high-probability ventilation/perfusion scan (V/Q scan), (3) had a systolic blood pressure on presentation ≥90 mmHg, (4) had troponin I (TnI) and/or B-natriuretic peptide (BNP) levels obtained within 24 h of admission, and (4) had an echocardiogram obtained within 48 h of admission.

Length of stay (LOS) was calculated for each eligible patient. Patients were later stratified, based on their LOS, into short LOS (≤2 days) and long LOS (>2 days) cohorts.

### 2.3. Methods and measurements

Based on detailed chart review, the patients’ demographics, medical comorbidities, risk factors, clinical presentation, vital signs, and initial workup were collected.

- Demographic parameters: age, gender, race and body mass index (BMI).
- Medical comorbidities: diabetes, hypertension, hyperlipidemia, smoking history, heart disease, lung disease, previous stroke, and renal impairment.
- Risk factors: immobilization (≥3 days), recent surgery (within the last 4 weeks), recent travel (≥4 h), recent estrogen therapy (within the last 3 months), concomitant documented deep-venous thrombosis (DVT), previous history of VTE, and history of cancer.
- Clinical features on presentation: chest pain, dyspnea, palpitations, syncope, cough, hemoptysis, wheezing, leg pain or swelling, altered mentation, and hemodynamic instability.
- Initial workup: complete blood count, renal functions, D-dimer, lactic acid and electrocardiogram (EKG), TnI, and BNP obtained within 24 h of admission and echocardiogram obtained within 48 h of admission.

Echocardiograms were evaluated for right ventricular (RV) dysfunction and were reported by a board-certified cardiologist.

A cutoff values for TnI of 0.10 ng/mL and for BNP of 100 pg/mL were predefined. Based on the available data, Bova score, GPS, PESI, and sPESI were determined for each eligible patient (Table 1).

**Table 1.** Clinical prognostic scores.

**1.1 Bova score**	
**Variables**	**Points**
SBP ≤100 mmHg	+2
Elevated cardiac troponin	+2
RV dysfunction	+2
Heart rate ≥110 beats/min	+1
The total score is calculated as the sum of the points assigned to each variable and is used to classify patients as Stage I, II, or III	
**Stages**	**Points**
Stage I	0–2
Stage II	3–4
Stage III	>4
Low/intermediate risk is defined by a score ≤4, those with a score >4 are considered high risk	
**1.2 Geneva prognostic score (GPS)**	
**Variables**	**Points**
Cancer	+2
Heart failure	+1
Previous DVT	+1
SBP <100 mmHg	+2
SaO_2_ < 90%	+1
Concomitant proximal DVT	+1
The total score is calculated as the sum of the points assigned to each variable. Low risk is defined by a score of ≤2.	
**1.3 Pulmonary embolism severity index (PESI)**	
**Variables**	**Points**
Age	Age in years
Male gender	+10
Cancer	+30
Heart failure	+10
Chronic lung disease	+10
Heart rate ≥110 beats/min	+20
SBP <100 mmHg	+30
Respiratory rate >30 breath/min	+20
Temperature <36°C/96.8°F	+20
Altered mental status	+60
SaO_2_ <90%	+20
The total score is calculated as the sum of the age in years and the points assigned to each variable and used to classify patients as class I, II, III, IV, or V.	
**Classes**	**Points**
Class I	≤65
Class II	66–85
Class III	86–105
Class IV	106–125
Class V	>125
Low risk is defined by a score of ≤85 or a risk class ≤II.	
**1.4 Simplified Pulmonary Embolism Severity Index (sPESI)**	
**Variables**	**Points**
Age >80 years	+1
Cancer	+1
Chronic cardiopulmonary disease	+1
Heart rate ≥110 beats/min	+1
SBP <100 mmHg	+1
SaO_2_ < 90%	+1
The total score is calculated as the sum of the points assigned to each variable. Patients with a score of 0 were determined to be low risk, while those with a score of 1 or more were considered high risk.	

SBP, systolic blood pressure; RV dysfunction, right ventricular dysfunction; DVT, deep-venous thrombosis; SaO_2_, oxygen saturation.

The primary outcome of this study was the LOS in days. This was subdivided into two cohorts: LOS of 2 days or less and LOS of more than 2 days. The secondary outcomes were the association between clinical and biochemical variables and LOS.

### 2.4. Statistical analyses

Statistical analyses were performed using statistical package for the social sciences (SPSS), version 24.0 (SPSS Inc., Chicago, IL, USA). Descriptive statistics were provided for all variables and presented as mean ± standard deviation for continuous variables and as number and percentage for categorical variables. Statistical tests of significance (Student’s *t* test or Mann–Whitney *U* test for continuous variables and Chi-square test or Fischer’s exact test for categorical variables) were conducted to assess the differences between the cohorts (short LOS versus long LOS). A two-tailed *p* value ≤.05 was regarded as statistically significant. Binary logistic regression analysis was used to identify predictors of LOS among the study population. Variables with a *p* value ≤.20 were included in multivariate logistic regression analysis. A multivariate model was made using the Enter-method. To interpret the results of multivariate analyses, *p* values ≤.05 were considered significant.

## 3. Results

### 3.1. Patient characteristics

After applying inclusion and exclusion criteria, a total of 79 patients were included in the study. The demographic and clinical characteristics of the patients are shown in Table 2. Seventeen patients (22%) had short LOS (≤2 days) and 62 (78%) had long LOS (>2 days).

**Table 2.** Demographic and clinical characteristics of patients at baseline.

Characteristics	All patients (*N* = 79)	Length of stay		*p* Value
		Short (≤2 days) (*N* = 17)	Long (>2 days) (*N* = 62)	
Age (years)	55.76 ± 17.330	48.18 ± 14.838	57.84 ± 17.489	0.041*
Age group				0.028*
<65 years	52 (65.8)	15 (88.2)	37 (59.7)	
≥65 years	27 (34.2)	2 (11.8)	25 (40.3)	
Gender				0.481
Male	29 (36.7)	5 (29.4)	24 (38.7)	
Female	50 (63.3)	12 (70.6)	38 (61.3)	
Comorbidities				
Diabetes	18 (22.8)	2 (11.8)	16 (25.8)	0.332
Hypertension	45 (57.0)	9 (52.9)	36 (58.1)	0.705
Hyperlipidemia	15 (19.0)	1 (5.9)	14 (22.6)	0.170
Smoking	19 (24.1)	4 (23.5)	15 (24.2)	1.000
Heart disease	17 (21.5)	2 (11.8)	15 (24.2)	0.338
Lung disease	26 (32.9)	2 (11.8)	24 (38.7)	0.036*
Stroke	12 (15.2)	3 (17.6)	9 (14.5)	0.714
Renal impairment	13 (16.5)	1 (5.9)	12 (19.4)	0.278
Number of comorbidities	2.09 ± 1.562	1.41 ± 1.372	2.27 ± 1.570	0.043*
Risk factors				
Immobilization	13 (16.5)	2 (11.8)	11 (17.7)	0.723
Recent surgery	4 (5.1)	0	4 (6.5)	0.572
Recent travel	4 (5.1)	3 (17.6)	1 (1.6)	0.030*
Recent estrogen therapy	6 (7.6)	2 (11.8)	4 (6.5)	0.604
Concomitant DVT	11 (13.9)	4 (23.5)	7 (11.3)	0.238
Previous VTE (DVT/PE)	13 (16.5)	1 (5.9)	12 (19.4)	0.278
Active cancer	6 (7.6)	0	6 (9.7)	0.331
Symptoms				
Chest pain	31 (39.2)	6 (35.3)	25 (40.3)	0.707
Dyspnea	52 (65.8)	12 (70.6)	40 (64.5)	0.640
Palpitations	13 (16.5)	2 (11.8)	11 (17.7)	0.723
Syncope	6 (7.6)	1 (5.9)	5 (8.1)	1.000
Cough	10 (12.7)	3 (17.6)	7 (11.3)	0.441
Hemoptysis	6 (7.6)	2 (11.8)	4 (6.5)	0.604
Wheezing	3 (3.8)	1 (5.9)	2 (3.2)	0.522
Leg pain/swelling	22 (27.8)	5 (29.4)	17 (27.4)	1.000
Altered mentation	1 (1.3)	0	1 (1.6)	1.000
Initial vital signs				
Heart rate (beats/min)	97.75 ± 20.465	89.71 ± 15.846	99.95 ± 21.136	0.067
Systolic blood pressure (mmHg)	136.33 ± 23.580	137.18 ± 17.802	136.10 ± 25.051	0.868
Oxygen saturation (%)	96.44 ± 3.957	97.18 ± 2.128	96.24 ± 4.318	0.392
Initial EKG				
Normal	39 (49.4)	10 (58.8)	29 (46.8)	0.379
Sinus bradycardia	3 (3.8)	1 (5.9)	2 (3.2)	0.522
Sinus tachycardia	14 (17.7)	2 (11.8)	12 (19.4)	0.722
Atrial arrhythmia	8 (10.1)	3 (17.6)	5 (8.1)	0.359
New RBBB	2 (2.5)	1 (5.9)	1 (1.6)	0.386
New inferior Q waves	4 (5.1)	0	4 (6.5)	0.572
New anterior ST-T changes	4 (5.1)	2 (11.8)	2 (3.2)	0.201
S_1_Q_3_T_3_	2 (2.5)	0	2 (3.2)	1.000
Troponin I (cutoff 0.10ng/mL)				
Negative	53 (67.1)	14 (82.4)	39 (62.9)	0.131
Positive	26 (32.9)	3 (17.6)	23 (37.1)	
B-natriuretic *peptide* (cutoff 100 pg/mL)				
Negative	26 (39.4)	7 (53.8)	19 (35.8)	0.234
Positive	40 (60.6)	6 (46.2)	34 (64.2)	
RV dysfunction	22 (27.8)	4 (23.5)	18 (29.0)	0.767
Bova score (points)	1.59 ± 1.971	0.88 ± 1.409	1.79 ± 2.066	0.094
Bova stage				0.164
Stage I	56 (70.9)	15 (88.2)	41 (66.1)	
Stage II	16 (20.3)	2 (11.8)	14 (22.6)	
Stage III	7 (8.9)	0	7 (11.3)	
PESI score (points)	77.28 ± 33.170	55.24 ± 16.758	83.32 ± 34.069	0.002*
PESI score				0.002*
≤85	55 (69.6)	17 (100)	38 (61.3)	
>85	24 (30.4)	0	24 (38.7)	
Simplified PESI				0.001*
Low risk	33 (41.8)	13 (76.5)	20 (32.3)	
High risk	46 (58.2)	4 (23.5)	42 (67.7)	
Geneva prognostic score (points)	0.73 ± 1.118	0.41 ± 0.712	0.82 ± 1.195	0.232
Geneva prognostic score				0.331
≤2	73 (92.4)	17 (100)	56 (90.3)	
>2	6 (7.6)	0	6 (9.7)	

Data are presented as mean ± SD or count (percentage). BMI, body mass index; DVT, deep-venous thrombosis; VTE, venous thromboembolism; PE, pulmonary embolism; RBBB, right bundle branch block; CTA, computed tomography angiography; V/Q scan, ventilation/perfusion scan; RDW, red cell distribution width; BUN, blood urea nitrogen; GFR, glomerular filtration rate; RV dysfunction, right ventricular dysfunction; LMWH, low-molecular-weight heparin; NOAC, novel oral anticoagulants; IVC, inferior vena cava; PESI, pulmonary embolism severity index.

*p value <.05 is considered statistically significant.

In our study population, 29 patients (37%) were males and 50 (63%) were females. Elderly patients (defined as age ≥65 years) comprised 34% of the study population compared to younger patients with age <65 years (66%). Majority of our patients (94%) were African-Americans. Age groups showed statistically significant difference in LOS; *χ*^2^ (1, *N* = 79) = 4.84, *p* = .028, with patients in short LOS being younger (*M* = 48.18, *SD* = 14.84) compared to long LOS (*M* = 57.84, *SD* = 17.49). However, gender did not show any statistically significant difference in LOS between the two groups; *χ*^2^ (1, *N* = 79) = 0.50, *p* = .481. In 86% of the patients, PE was confirmed by pulmonary CTA, while V/Q scan was utilized when renal impairment precluded the use of contrast in 14% of cases.

Hypertension was the most common comorbidity present on more than half of the patients (57%) followed by lung disease (33%), smoking (24%), and diabetes (23%), in descending order of prevalence. Patients with long LOS were more likely to have more comorbidities than patients with short LOS, but no statistically significant differences were found between the two cohorts for each comorbidity evaluated with the exception of lung disease, which was more common in long LOS group (39%) compared to short LOS group (12%), *χ*^2^ (1, *N* = 79) = 4.39, *p* = .036. In general, there was a statistically significant difference in the total number of comorbidities between patients with LOS ≤2 days and >2 days (*M* = 1.41, *SD* = 1.37 and *M* = 2.27, *SD* = 1.57, respectively), *t* (77) = 2.06, *p* = .043.

The most common risk factors were recent immobilization (≥3 days), history of previous VTE, and concomitant proximal DVT diagnosed at the time of admission representing 17%, 17%, and 14%, respectively. Recent surgery and associated cancer were only present in long LOS group. On the other hand, there was a statistically significant difference in the history of recent travel as a risk factor between the two groups and was associated with short LOS, with most patients with history of travel (18%) being discharged within 2 days of admission (*p* = .030).

Dyspnea was the most frequent presenting complaint (66%) followed by chest pain (39%), leg swelling and/or pain (28%), and palpitations (17%). Very few patients presented with altered mentation (1%), but they had long LOS. There were no statistically significant differences in vital signs between the two groups: pulse rate, systolic blood pressure, or oxygen saturation (*t*(77) = 1.86, *p* = .067; *t*(77) = 0.17, *p* = .868; and *t*(77) = 0.86, *p* = .392, respectively).

Half of our patients (49%) had a normal EKG on presentation. EKG abnormalities when present were mostly sinus tachycardia (18%). Few patients had atrial arrhythmias (10%), new inferior Q waves or anterior ST-T changes (5%), sinus bradycardia (4%), S_1_Q_3_T_3_ pattern (3%), and new RBBB (3%). Both biochemical marker values, TnI and BNP, were more likely to be positive in long LOS than in short LOS, but no statistically significant difference was found (*χ*^2^(1, *N* = 79) = 2.29, *p* = .131 and *χ*^2^(1, *N* = 66) = 1.42, *p* = .234, respectively). Patients with RV dysfunction accounted for 28% and more than three-quarters (82%) were in long LOS group (*p* = .767).

Most patients were treated with low-molecular-weight heparin (LMWH) and/or warfarin (39% and 47%, respectively). Around 35% received novel oral anticoagulants (NOACs) and only three patients received inferior vena cava filters for secondary prevention of PE. Only one patient had serious bleeding as a complication of anticoagulation during admission. Most patients were safely discharged (96%). In-hospital mortality was 4%, occurring only in the long LOS group. Among all patients, 41% required ICU stay.

Among the clinical prognostic scores, both PESI (original and simplified) scores showed statistically significant difference between short and long LOS (*χ*^2^(1, *N* = 79) = 9.45, *p* = .002 and *χ*^2^(1, *N* = 79) = 10.72, *p* = .001, respectively). On the other hand, both Bova and GPS scores showed no statistically significant difference in mean between the two groups (*U* = 395.50, *Z* = −1.67, *p* = .094; *U* = 438.50, *Z* = −1.20, *p* = .0232; respectively).

### 3.2. Predictors of length of stay

Binary logistic regression models revealed a PESI score that significantly predicted long LOS (*OR* 1.067, 95% *CI* [1.001, 1.137], *p* = .048), even when adjusted for age, presence of lung disease, and the number of comorbidities. Similarly, sPESI also significantly predicted long LOS (*OR* 0.223, 95% *CI* [0.050, 0.999], *p* = .050), adjusted for age group, the presence of lung disease, and the number of comorbidities. Bova score was not a statistically significant predictor of LOS. Additionally, lung disease was not a statistically significant predictor of prolonged hospital stay in both regression models tested.

### 3.3. Optimal cutoff of prediction

**Figure 1.**
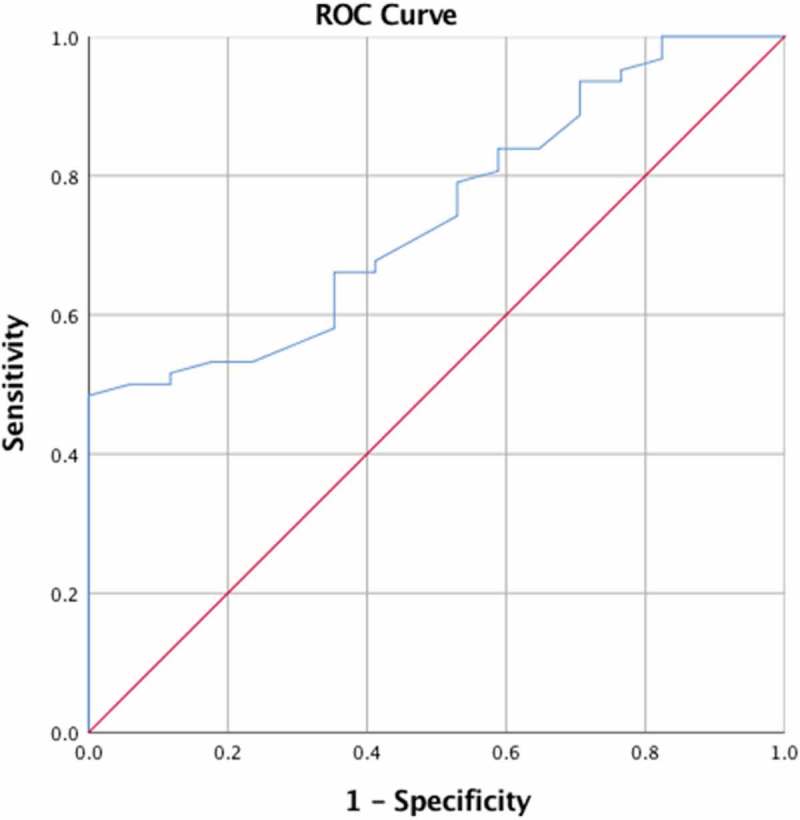
ROC curve with AUC to test the effectiveness of PESI score as a predictor of prolonged LOS.

The calculated receiver operating characteristic (ROC) analysis for PESI score predicting prolonged hospital showed an area under the curve (AUC) of .743 with a *p* value of .002 for differentiation. A PESI score cutoff value of 50.00 had a sensitivity and a specificity of 84% and 41%, respectively (Figure 1).

## 4. Discussion

Acute PE is one of the leading causes of morbidity and mortality [1]. During the acute phase, it is of supreme importance to stratify the severity of the disease because it may help guide decision-making in terms of appropriate setting of treatment. In a study conducted by Wang et al. [11], LRPE patients with a short LOS had better clinical outcomes than those with long LOS which was complicated with a higher rate of hospital-acquired morbidities. Our investigation about the association between the parameters and LOS is unique as it allowed us to determine the factors which lead to prolonged LOS.

The mortality of acute PE can be predicted with myocardial injury, RV dysfunction, and other biochemical parameters. Meta-analysis of five prospective studies of hemodynamically stable PE demonstrated that 44% of patients had RV dysfunction and a higher mortality rate with a sensitivity of 70%, a positive predictive value of 58%, and a negative predictive value of 60% for prediction of mortality [12]. In another study, the prognostic value of echocardiogram in hemodynamically stable PE patients was moderate [13]. In our study, 29% of patients had RV dysfunction, of which about (82%) belonged to long LOS group. However, these results were not statistically significant (*p* = .767). Yet, this finding was limited by its operator dependence, a relatively small sample size, and lack of reference echocardiograms for comparison prior to diagnosis of RV dysfunction.

Higher values of cardiac biomarkers (TnI and BNP) were noted in patients who belonged to long LOS when compared with short LOS group, but no statistically significant difference was found between the two groups, as well. Elevated cardiac biomarkers have been linked with an increased risk of mortality during the acute phase of PE [14]. Furthermore, Becatinni et al. published meta-analysis which supported the fact that troponin I and/or T elevations were associated with higher medical conditions and mortality even in the subgroup of low-risk and hemodynamically stable patients [15]. Nevertheless, large-scale investigations are clearly necessary to define the precise correlation of cardiac biomarkers and its association with duration of in-hospital stay for patients with an acute PE.

PESI, the most extensively validated prognostic model for acute PE, stratifies patients into five classes (Class I–V) based on 11 clinical parameters with different prognostic weights. Similar to the above parameters, PESI score was initially devised to predict mortality and to identify low-risk patients who may be candidates for outpatient treatment or abbreviated hospital stay [16]. sPESI, with similar prognostic accuracy, reduces complexity by including 6 of the 11 original PESI variables [7], and we aimed to analyze the usefulness of PESI and sPESI scores to predict LOS in normotensive patients with acute PE. Our investigation showed that a PESI score of <85 points and a sPESI score of 0 (low-risk class) identified patients at low risk and accurately predicted short LOS. Other factors that may result in prolonged LOS are the presence of lung disease and the total number of comorbidities. The calculated effectiveness in predicting prolonged LOS showed *AUC* of 0.743 (*p* = .002) with a sensitivity and specificity of 84% and 41% when the diagnostic cutoff was further lowered to score of 50, respectively. Based on these findings, physicians should be more comfortable with outpatient treatment especially when home services are adequate which is further supported by recently updated guidelines [12].

In summary, the results of this study showed that PESI score is a major predictor in LRPE patients to define short LOS. The risk stratification is of utmost importance as it can safely categorize patients eligible for short-term hospital stay which in turn reduces disease’s clinical and financial burden. Even though cardiac biomarkers and RV dysfunction are predictors of mortality, their role in defining LOS is yet to be established.

## 5. Conclusion

PE severity stratification is essential in determining LOS. Several clinical, laboratory, and imaging parameters have been used to assess PE severity. Physicians are expected to take into consideration all these variables in their management plan. PE predictors of LOS might help to assess PE severity and may effectively help to reduce LOS, health care utilization, and cost. However, more studies with a larger sample size are needed to come up with a more accurate tool for assessment of PE severity, which can assist physicians to take decision prospectively about hospital stay for optimal treatment of PE patients.

## 6. Limitations

This study is subject to the limitations inherent in its retrospective design. We conducted a review of 149 charts over the period of 2 years, and only 79 patients were eligible to be included in this study as per our selection criteria. As a result, the number of patients is relatively small, which potentially affects the statistical power of the study.
